# Ameliorative Effect of *Olea europaea* Leaf Extract on Cisplatin-Induced Nephrotoxicity in the Rat Model

**DOI:** 10.1155/2023/2074498

**Published:** 2023-07-18

**Authors:** Doa'a Ibrahim, Abdulsalam Halboup, Mohammed Al Ashwal, Amani Shamsher

**Affiliations:** ^1^Department of Clinical Pharmacy and Pharmacy Practice, Faculty of Pharmacy, University of Science and Technology, Sana'a, Yemen; ^2^Discipline of Clinical Pharmacy, School of Pharmaceutical Sciences, Universiti Sains Malaysia, Penang, Malaysia; ^3^Department of Pharmacology, Faculty of Pharmacy, University of Science and Technology, Sana'a, Yemen; ^4^Department of Histopathology, University of Science and Technology Hospital, Sana'a, Yemen

## Abstract

**Background:**

*Olea europaea* leaf extract (OELE) has potential health benefits and protects against cytotoxicity. This study investigated the possible ameliorative effect of OELE on cisplatin-induced nephrotoxicity in rats.

**Methods:**

Rats were assigned into six groups; two groups received 150 mg/kg or 300 mg/kg of OELE, one group received a single dose of cisplatin (6 mg/kg) IP on the first day of the experiment, two groups received a single dose of cisplatin 150 mg/kg or 300 mg/kg of OELE on the first day then starting from the fifth day for 10 consecutive days, and one group acted as a control. *Results and Conclusion*. The findings showed that cisplatin-induced nephrotoxicity was evidenced by a significant increase in serum creatinine blood urea nitrogen (BUN) and a significant decrease in estimated creatinine clearance and potassium level, which corresponded with the alterations in the histopathology of the renal tissue. OELE significantly ameliorated the nephrotoxic effects of cisplatin as dose-dependent.

## 1. Introduction

Cisplatin is a platinum-derived chemotherapeutic agent frequently used as a standard antineoplastic drug for treating different types of malignancies in the lung, bladder, cervix, ovaries, and testes [1, 2]. Cisplatin is eliminated through the renal system and accumulates in the renal tubules [3], leading to increased cisplatin concentrations in the proximal tubule cells that are five times higher than the serum level [4]. Therefore, it is limited to patients with creatinine clearance greater than 60 ml/minute [5]. Although cisplatin induces various dose-limiting adverse effects, such as neurotoxicity, ototoxicity, gastrotoxicity, myelosuppression [2, 3], cardiotoxicity [6], testicular toxicity [7], and hepatorenal toxicity [8], nephrotoxicity is the most prominent adverse effect encountered in clinical practice. Nephrotoxicity induced by cisplatin is often seen after 10 days of cisplatin administration and can present with various types of symptoms, including acute kidney injury (AKI), renal tubular acidosis, hypomagnesemia, hyperuricemia, and hypocalcemia [9]. Clinical evidence has revealed that one-third of patients experience AKI after cisplatin administration, along with increases in blood urea nitrogen (BUN), serum creatinine, reduced glomerular filtration rate (GFR), and disturbed electrolytes [10, 11].

*Olea europaea*, also known as the olive tree, is an evergreen tree that is indigenous to the Mediterranean region, where it is widely utilized as traditional medicine for a variety of illnesses [12]. Among the different parts of the *Olea europaea* tree, the leaves have the highest antioxidant and scavenging capacity [13]. The extracted leaves of *Olea europaea* have drawn attention since they have phenolic compounds that are linked to various pharmacological activities [14]. Several *in vitro* and *in vivo* studies have demonstrated wide range of health benefits of the extracted *Olea europaea* leaves, such as cytotoxic activity against human breast cancer cells [15], an antiproliferative effect on leukemia cells [16, 17], an antiarrhythmic effect [18], a hypotensive effect [19], an anti-HIV effect [20], and an antimalaria effect [21]. The wide range of these pharmacological activities is mostly linked to oleuropein and its bioactive byproduct, hydroxytyrosol, which comprises the major active compound in olive leaves [22]. This endogenous compound in OELE possesses empower antioxidant activities [14] that provide sufficient protection against the reactive oxygen species (ROS) resulted from cell damage or burst [22]. Several studies on the antioxidants of hydroxytyrosol and oleuropein have been performed. A recent study showed that both oleuropein and hydroxytyrosol have hypolipidemic and hepatoprotective effects against high-fat diet-induced metabolic disorders [23]. The nephroprotective effect of *Olea europaea* leaf extract (OELE) has been reported on diclofenac-induced hepatotoxicity and kidney damage [24] and gentamicin-induced nephrotoxicity [25, 26]. In addition, the ameliorative effect of the extracted *Olea europaea* leaves has been reported on nephrotoxicity induced by cyclosporin [27] and cyclophosphamide in an animal model [28]. In human embryonic renal epithelial “GP-293” cells, oleuropein has exhibited a protective effect against cisplatin-induced cellular toxicity [29]. However, the nephroprotective effect of different doses of OELE against cisplatin-induced nephrotoxicity in a rat model has not been evaluated yet. Thus, the aim of this study is to evaluate the ameliorative effect of different doses of the *Olea europaea* leaf extract against cisplatin-induced nephrotoxicity in rat models. It also aimed to study the effect of the extracted leaves and/or cisplatin on the body's and organs' weight and potassium level as a major complication of acute kidney injury.

## 2. Materials and Methods

### 2.1. Animals and Treatment

Adult healthy Wistar male rats weighing 160 ± 20 g were brought from the Biology Department, Sana'a University. Animals were housed in a group of five in each metal cage under hygienic conditions at a temperature of 23 ± 2°C, relative humidity of 50–60%, and 12 h of light/dark cycles. All experiments were carried out in the light cycle according to the protocol of the Animal Care and Use Committee, National Research Council (Washington, 2011). Animals were identified by tail labeling and provided with standard rodent food with free access to water ad libitum. All procedures were reviewed and approved by the ethical committee at the University of Science and Technology (reference number: EAC/UST231). Before starting the experiments, the animals were kept for two weeks to ensure acclimatization.

### 2.2. Drugs and Natural Products

A cisplatin vial (1 mg/mL; Zuviplat®) in the clinical formulation was purchased from Zuvius Lifesciences Pvt. Ltd. (Mumbai, India).

The fresh leaves of *Olea europaea* were collected from the olive trees in the Sana'a region, Yemen, at the beginning of September 2016. Identification and authentication of the collected plant specimen were done by a botanist from the Faculty of Sciences, Department of Botany, Sana'a University. A voucher specimen was deposited in the Yemeni National Herbarium, Sana'a University, Yemen, with the collection number (Herbarium number: 718).

### 2.3. Induction of Acute Kidney Injury (AKI)

Acute kidney injury (AKI) was induced in rats by intraperitoneal administration of a single cisplatin dose (6 mg/kg; Zuviplat®) according to previous studies [30–32]. After the induction of AKI, the rat had free access to food and water.

### 2.4. Plant and Sample Preparation

The fresh leaves of *Olea europaea* were collected from the olive trees in the Sana'a region at the beginning of September 2016. The leaves were thoroughly washed using tap water to remove dust and then dried at room temperature in a dark place for 14 days. The dried leaves were ground using a blender to a fine powder and then packaged in a glass container and stored at 4°C for further analysis.

### 2.5. Preparation of *Olea europaea* Leaf Extraction

The air-dried leaves (170 g) were extracted with 95% ethanol (1 : 25 w⁄v) using a Soxhlet apparatus for 24 hours and concentrated using a rotary evaporator (Büchi®, Rotavapor R-200) at 40°C. The resulting extract, 120 g (a yield of 70.6%), was stored at 15–20°C in sealed desiccators, and the drying process was carried out in closed and controlled equipment using a Labconco freeze dryer to improve the quality of the final product [33]. The required amount of the extracted *Olea europaea* leaves was dissolved in normal saline and administered to the animals by oral gavage according to their body weight [33, 34].

### 2.6. Phytochemical Screening

Screening for the presence or absence of flavonoids, alkaloids, anthraquinones, saponins, tannins, glycosides, and terpenoids was qualitatively carried out as described in [35, 36].

### 2.7. Acute Oral Toxicity Study

An acute oral toxicity study of the extracted leaves was carried out according to the OECD 425 guidelines and methods of Jaykaran et al. [37]. After a two-week acclimatization period, twelve rats weighing 180 ± 20 g were randomly assigned into three groups, with four rats in each group, as follows: Group I served as a control group, which has free access to rodent food and water; Group II was given 2 g/kg of the extract of *Olea europaea* leaves; and group III was given 5 g/kg of the same extract for 72 hours. The extract was previously dissolved in water and given to the animals by oral gavage (once a day) for 72 hours. Rats from the two treatment groups were observed individually for any sign of toxicity after the treatment, as described in [37, 38].

### 2.8. Study Design

Thirty male Wistar rats with age 2-3 months, weighing 160 ± 20 g, were randomly assigned into six groups, with five rats per group, as follows: the control group, which had free access to water for 15 days; the cisplatin (CIS) group was given a single intraperitoneal dose (6 mg/kg) of cisplatin and utilized as a positive control; two groups (treated groups: CIS/OELE-150/kg and CIS/OELE-300/kg) were given a single dose of intraperitoneal cisplatin (6 mg/kg) on the first day, and then 150 mg/kg and 300 mg/kg of the extracted leaves of *Olea europaea*, starting from the 5th day to the 15th day; two groups were given 150 mg/kg and 300 mg/kg (OELE-150/kg, and OEL-300/kg) of OELE for 15 days according to the method [28] and utilized as a negative control. On the sixth day of the experiment, blood samples were withdrawn for nephrotoxicity evaluation, and at the end of the experiment (15th day), blood samples were collected using a heart puncture under halothane anesthesia, and both kidneys were dissected for histopathological study [28].

### 2.9. Biochemical Parameters

Kidney function tests, including serum creatinine (Chema Diagnostica, Monsano (AN)-Italy), blood urea nitrogen (Chema Diagnostica, Monsano (AN)-Italy), estimated creatinine clearance, and potassium level (Spinreact, Ctra.Santa Coloma, Girona, Spain), were evaluated using the COBAS 6000 analyzer.

### 2.10. Creatinine Clearance Estimation

Creatinine clearance estimation was carried out using a recently published model. This model depends on plasma creatinine and the weight of rats for the estimation of creatinine clearance with high accuracy, regardless of other factors such as the age of the rats, comorbidities, and treatment. The model was validated and found to have congruent results with the measured creatinine clearance. This model was recently released as a web-based calculator (https://idal.uv.es/aclara) to facilitate its use [39].

### 2.11. Histopathology of the Kidney

The animals were sacrificed by decapitation under light anesthesia using halothane in desiccator specialized for anesthesia on the same day of blood collection. Both kidneys were dissected immediately. The organs were washed using normal saline water and weighed. Both kidneys were preserved in 10% phosphate buffer and formalin for histopathologic investigations [40]. Kidneys were embedded in paraffin wax and chopped into small serial pieces, cut at 4 *μ*m using a rotary microtome. They were stained with hematoxylin and eosin and then observed under a light microscope (Leica Microsystems, Germany) by a histopathologist in a blind manner.

Different histopathological alterations, including tubular necrosis, proteinaceous casts, and medullary congestion, were examined. The severity of these alterations was categorized as follows: normal renal tissue indicating no damage, mild damage characterized by the presence of unicellular or patchy isolated damage, moderate damage representing renal damage of less than 25% but greater than the mild, severe damage representing renal damage between 25% and 50%, and very severe damage indicating damage exceeding 50% of the renal tissue [40].

### 2.12. Statistical Analyses

All data were expressed as means ± standard error of the means (SEM). Statistical analysis was carried out using GraphPad Prism version 7.0 (GraphPad Software, Inc., San Diego, USA). Comparisons of biomarkers among the different study groups were evaluated by one-way ANOVA followed by Tukey's posttest. Two-way ANOVA followed by the Bonferroni posttest was also used to compare groups at different time points (the 6th day and the last day of the experiment as repeated-measures factors). The results of this study are considered significant at a *P* value less than 0.05.

## 3. Results

### 3.1. Phytochemical Screening

Phytochemical screening tests of OELE showed the presence of flavonoids, anthraquinones, tannins, saponins, alkaloids, and terpenoids.

### 3.2. Findings of the Acute Oral Toxicity Study

Administration of 2 g/kg and 5 g/kg of the extracted leaves to different groups of rats for 72 hours did not show signs of poisoning such as lacrimation, hair erection, convulsion, coma, or death, a significant difference in the body weights, compared to the control group. The findings showed no significant difference in the hemoglobin level (Hb) or white blood cell (WBC) and other biochemical studies compared with the control group. These findings indicated that this plant has LD50 greater than 5 g/kg.

### 3.3. Results of the Main Experiment

#### 3.3.1. Effect of OELE and/or Cisplatin on Serum Creatinine Levels in Rats

**Figure 1:**
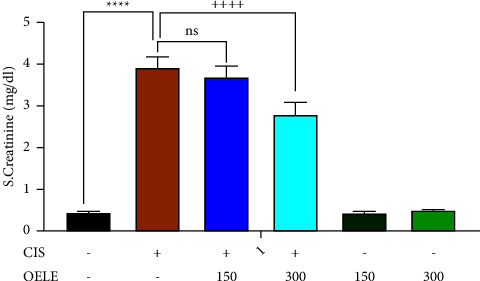
Effect of OELE on day 6 of cisplatin administration. Notes: each bar is expressed as mean ± SEM. Cisplatin vs. control group: ^*∗∗∗∗*^*P* < 0.0001, ^*∗∗∗*^*P* < 0.001, ^*∗∗*^*P* < 0.01, and ^*∗*^*P* < 0.05; CIS vs. (CIS/OELE-150 and CIS/OELE-300): ^++++^*P* < 0.0001, ^+++^*P* < 0.001, and ^++^*P* < 0.01, ^+^*P* < 0.05. CIS: cisplatin; OELE: *Olea europaea* leaf extract; ns: nonsignificant; *n* = 5 rats/group.

**Figure 2:**
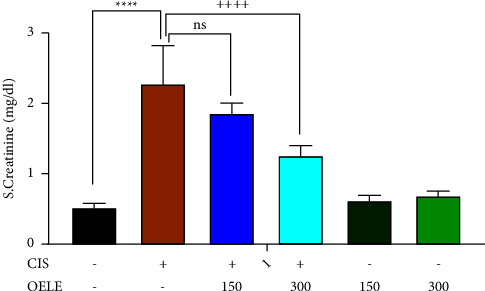
Effect of OELE on day 15 of cisplatin administration. Notes: each bar is expressed as mean ± SEM. Cisplatin vs. control group: ^*∗∗∗∗*^*P* < 0.0001, ^*∗∗∗*^*P* < 0.001, ^*∗∗*^*P* < 0.01, and ^*∗*^*P* < 0.05; CIS vs. (CIS/OELE-150 and CIS/OELE-300): ^++++^*P* < 0.0001, ^+++^*P* < 0.001, ^++^*P* < 0.01, and ^+^*P* < 0.05. CIS: cisplatin; OELE: *Olea europaea* leaf extract; ns: nonsignificant; *n* = 5 rats/group.

A single intraperitoneal dose of cisplatin (6 mg/kg) administered on the first day of the experiment resulted in persistent AKI on the sixth and fifteenth days of the experiment, as evidenced by a significant increase in serum creatinine levels (*P* < 0.0001; Figure 1) compared to the control group. However, the administration of 300 mg/kg of ethanol-extracted* Olea europaea* leaves on day 5 of the experiment showed a significant improvement (*P* < 0.0001) in serum creatinine when compared with the cisplatin-treated group. Similar findings were observed when a second blood sample was collected on the 15th day of the experiment. The finding revealed persistent kidney damage on the 15th day of cisplatin administration, as evidenced by a persistent increase in the serum creatinine levels (*P* < 0.0001), compared with the control group, as shown in Figure 2. However, continuous administration of 300 mg/kg of ethanol-extracted *Olea europaea* leaves showed a significant (*P* < 0.0001) improvement in serum creatinine levels compared with the cisplatin-treated group. On the other hand, the lower dose (150 mg/kg) of the extracted *Olea europaea* leaves did not show significant improvement in the levels of serum creatinine at the two time points (the 6th and the 15th days) of the experiment when compared with the cisplatin-treated group. Normal rats receiving either 150 mg/kg or 300 mg/kg of the extracted leaves alone did not show any alteration in serum creatinine levels compared to the control group.

The effect of the time factor and continuous administration of the extracted leaves on serum creatinine is shown in Table 1. On the 15th day, all groups treated with cisplatin (cisplatin, CIS/OELE-150/kg, and CIS/OELE-300/kg) exhibited a significant decrease (*P* < 0.0001) in the mean serum creatinine levels compared to the levels observed on the 6th day of the experiment.

#### 3.3.2. Effect of OELE and/or Cisplatin on Urea Levels in Rats

**Figure 3:**
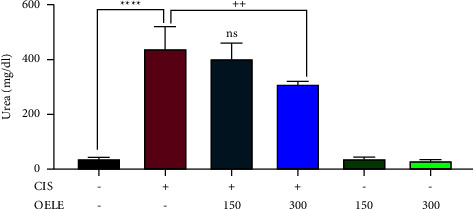
Changes in urea levels on day 6 of the experiment. Notes: each bar is expressed as mean ± SEM. Cisplatin vs. control group: ^*∗∗∗∗*^*P* < 0.0001; CIS vs. (CIS/OELE-150 and CIS/OELE-300): ^++^*P* < 0.01. CIS: cisplatin; OELE: *Olea europaea* leaf extract; ns: nonsignificant; *n* = 5 rats/group.

**Figure 4:**
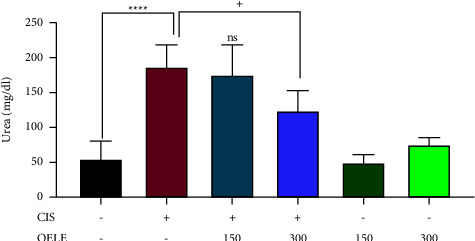
Changes in urea levels on day 15 of the experiment. Notes: each bar is expressed as mean ± SEM. Cisplatin vs. control group: ^*∗∗∗∗*^*P* < 0.0001; CIS vs. (CIS/OELE-150 and CIS/OELE-300): ^+^*P* < 0.05. CIS: cisplatin; OELE: *Olea europaea* leaf extract; ns: nonsignificant; *n* = 5 rats/group.

The ameliorative effect of different doses of OELE against cisplatin-induced nephrotoxicity was also evaluated by measuring blood urea levels on the 6th and 15th days of the experiment. Administration of cisplatin on day one resulted in AKI, as evidenced by the significant (*P* < 0.0001) increase in urea levels on both 6th and 15th days of the experiment when compared with the control group. However, continuous administration of 300 mg/kg of the extracted leaves by oral gavage on the 6th day resulted in a significant improvement in kidney function on the 6th (^++^*P* < 0.01) and 15th (^+^*P* < 0.05) days of the experiment when compared with the cisplatin-treated group. However, the lower dose of the extract (150 mg/kg) did not show a significant improvement in the urea levels on the 6th (*P* > 0.05) and 15th (*P* > 0.05) days of the experiment when compared with the cisplatin-treated group, as shown in Figures 3 and 4.

Table 2 presents the impact of cisplatin and/or extracted leaves on urea levels at two different time points. On the 15th day, all the groups treated with cisplatin (cisplatin, CIS/OELE-150, and CIS/OELE-300) exhibited a significant decrease (*P* < 0.0001) in the mean blood urea levels compared to the levels observed on the 6th day of the experiment.

#### 3.3.3. Effect of Cisplatin with/without the Extracted Leaves of *Olea europaea* on Creatinine Clearance

**Figure 5:**
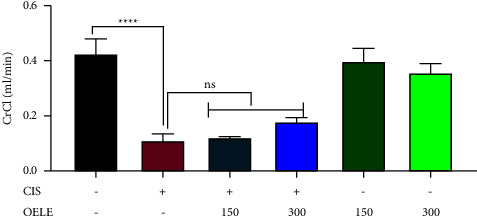
Estimated creatinine clearance on the last day of the experiment. Notes: each bar is expressed as mean ± SEM. Cisplatin vs. control group: ^*∗∗∗∗*^*P* < 0.0001; CIS vs. (CIS/OELE-150 and CIS/OELE-300): ^++^*P* < 0.01. CIS: cisplatin; OELE: *Olea europaea* leaf extract; ns: nonsignificant; *n* = 5 rats/group.

As shown in Table 3 and Figure 5, cisplatin-treated groups showed a significant reduction in the estimated creatinine clearance compared to the control group (*P* value <0.0001). Administration of high doses of the extracted *Olea europaea* leaves showed nonsignificant improvement in the estimated creatinine clearance. However, the low dose of the plant did not show any improvement in the estimated creatinine clearance.

#### 3.3.4. Effect of OELE and/or Cisplatin on Electrolytes in Rats

**Figure 6:**
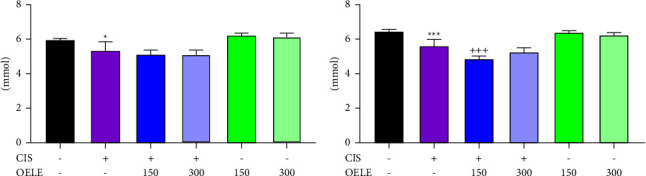
(a) Changes in serum potassium level on day 6 of the experiment. Notes: each bar is expressed as mean ± SEM. Cisplatin vs. control group: ^*∗*^*P* < 0.05; CIS vs. (CIS/OELE-150 and CIS/OELE-300). CIS: cisplatin; OELE: *Olea europaea* leaf extract; ns: nonsignificant; *n* = 5 rats/group. (b) Changes in serum potassium level on day 15 of the experiment. Notes: each bar is expressed as mean ± SEM. Cisplatin vs. control: ^*∗∗∗*^*P* < 0.001; CIS vs. (CIS/OELE-150 and CIS/OELE-300): ^+++^*P* < 0.001. CIS: cisplatin; OELE: *Olea europaea* leaf extract; ns: nonsignificant; *n* = 5 rats/group.

**Figure 7:**
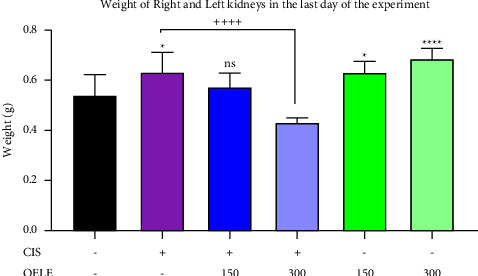
Weight of the right and left kidneys of the rats at the end of the experiment. Notes: each bar is expressed as mean ± SEM. Cisplatin vs. control group: ^*∗∗∗∗*^*P* < 0.001 and ^*∗*^*P* < 0.05; CIS vs. (CIS/OELE-150 and CIS/OELE-300): ^++++^*P* < 0.001. CIS: cisplatin; OELE: *Olea europaea* leaf extract; ns: nonsignificant; *n* = 5 rats/group.

The data illustrated a significant reduction in the serum potassium levels in the cisplatin-treated group on the 6th day (*P* < 0.05; Figure 6). Also, a further reduction was detected on the 15th day (*P* < 0.001; Figure 7) of the experiment compared with the control group. However, the administration of 150 mg/kg or 300 mg/kg of OELE for the cisplatin-treated group resulted in a further reduction in potassium levels on the 6th and 15th days of the experiment, and the lower dose of OELE resulted in a significant reduction in potassium levels on the 15th day compared with the cisplatin group. No significant differences in the potassium levels were observed when the rats received different doses of the extracted leaves of *Olea europaea* alone compared with the control group on both 6th and 15th days of the experiment.

The effect of the time factor on the potassium levels was studied in the presence of other variables. As shown in Table 4, the findings showed no significant (*P* > 0.05) differences in the potassium levels between the different groups on the 6th and 15th days of the experiment.

#### 3.3.5. Effect of OELE and/or Cisplatin on Rats' Weight

As shown in Table 5, regular increases in the body weight of rats were observed in the control group. There was a significant (*P* < 0.01) increase in the weight of rats between day 1 and day 15 of the experiment. However, all the cisplatin-treated groups (cisplatin, CIS/OELE-150, and CIS/OELE-300) showed a reduction in weight on day 15 compared with the weight on day 1. This effect was significant (*P* < 0.01 and *P* < 0.0001) when cisplatin was concurrently administered with either 300 mg/kg or 150 mg/kg of the extract, respectively.

Oral administration of 300 mg/kg of OELE alone prevented weight gain, while a lower dose (150 mg/kg) of the same extract did not prevent weight gain as appeared on day 15 when compared with the weight of the same rats on day 1.

#### 3.3.6. Effect of Cisplatin and/or OELE on the Weight of Rats' Kidneys on the Last Day of the Experiment

At the end of the treatment period, a significant increase (*P* < 0.05) in the weight of both kidneys was detected in the cisplatin-treated group when compared with the control group. In addition, normal rats that received different doses of the extract for 15 days showed a significant increase in the weight of the kidneys at a higher dose (300 mg/kg; *P* < 0.0001) and a lower dose (150 mg/kg; *P* < 0.05) of the extract compared with the control group. However, administration of a higher dose (300 mg/kg) of the extracted leaves to the cisplatin-treated group resulted in a significant (*P* < 0.01) reduction in the weight of the kidneys on the last day of the treatment period compared with the cisplatin-treated group. No significant (*P* > 0.05) difference in the weight of the kidneys was detected when the cisplatin-treated rats received a lower dose (150 mg/kg) of the same extract, as shown in Figure 7.

### 3.4. The Results of the Histopathologic Examination

#### 3.4.1. The Effect of Cisplatin and/or OELE on the Histology of the Kidney

**Figure 8:**
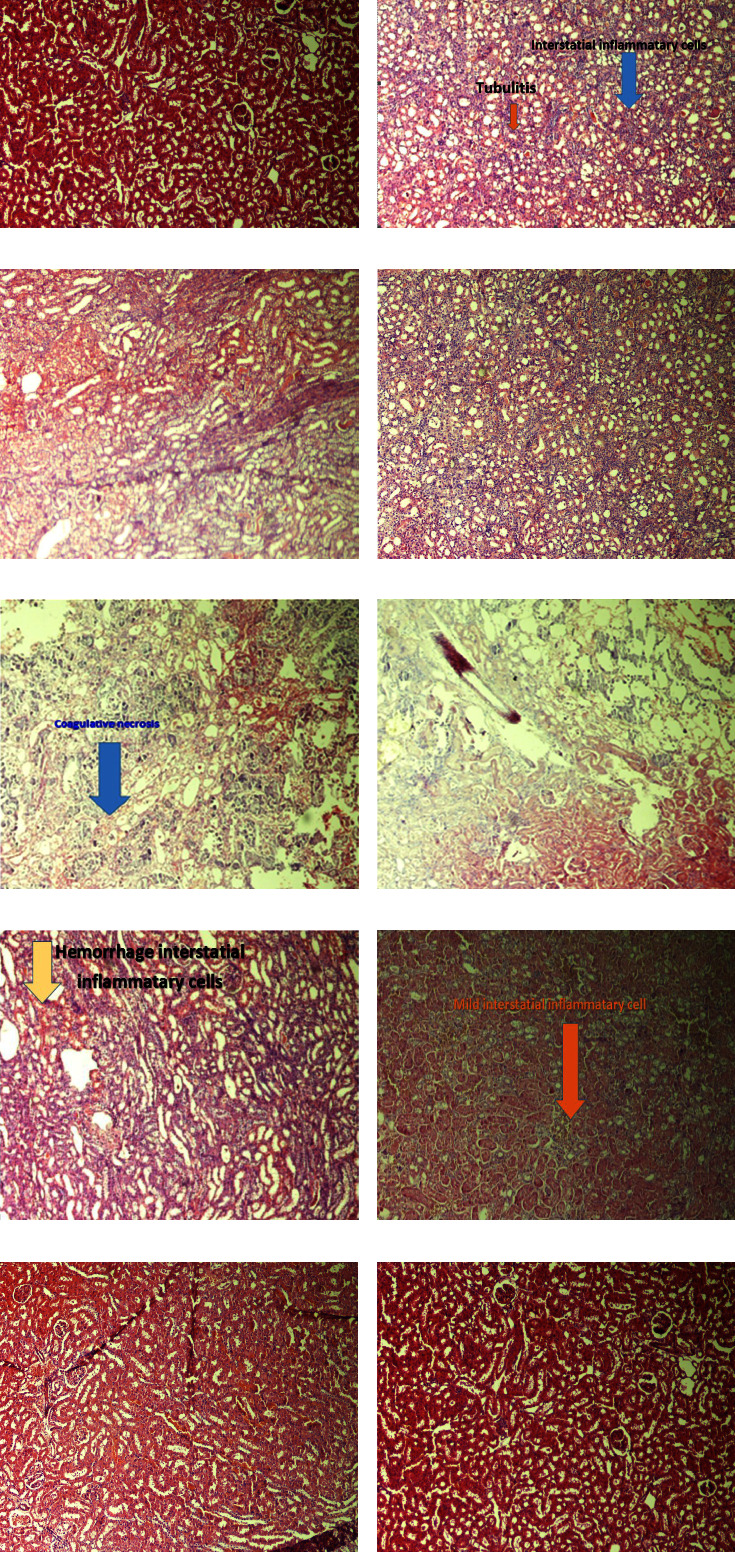
Effect of cisplatin and/or OELE on kidney histology of experimental groups: (a) section from the normal control group show normal renal tissues, (b, c) tissue sections from cisplatin-treated rats show renal tissues with moderate interstitial inflammatory cells mainly lymphocytes with multiple foci of tubulitis, (d–f) tissue sections from cisplatin-treated rats show renal tissues with multiple foci of coagulative necrosis and calcification, (g) tissue section from cisplatin-treated rats which received a dose of 150 mg/kg of the extracted *Olea europaea* leaves shows renal tissues with mild and hemorrhage interstitial inflammatory cells, (h) tissue section from cisplatin-treated rats which received a dose of 300 mg/kg of the extracted *Olea europaea* leaves shows renal tissues with mild interstitial inflammatory cells, (i) tissue section from normal rats received a Doe of 300 mg/kg of the extracted *Olea europaea* leaves alone shows normal renal tissues, and (j) tissue section from normal rats received a dose of 150 mg/kg of the extracted *Olea europaea* leaves shows normal renal tissues.

Histopathological study showed several structural changes and alterations in the hematoxylin and eosin-stained kidney section of cisplatin-treated rats. These alterations were shown as moderate interstitial inflammatory cells mainly lymphocytes with multiple foci of tubulitis along with multiple foci of coagulative necrosis and calcification, as shown in Figures 8(b)–8(f). However, administration of 300 mg/kg of the extracted *Olea europaea* leaves ameliorated cisplatin-induced interstitial inflammation of the renal tissue to the mild stage, as shown in Figure 8(h). Moreover, the lower dose (150 mg/kg) of the extracted leaves in cisplatin-treated rats revealed amelioration of the interstitial inflammation but induced hemorrhage, as shown in Figure 8(g).

Control rats and normal rats received either 300 mg/kg or 150 mg/kg of OELE and showed normal morphology and histological structure of glomeruli and renal tubule, as shown in Figures 8(a), 8(i), and 8(j).

## 4. Discussion

The renal system plays a crucial role in the regulation of blood volume and the excretion of toxic materials from the body. Kidneys have a high capacity level for drug uptake, making them vulnerable to drug toxicity and injury. An efficient therapeutic agent to attenuate cisplatin-induced nephrotoxicity is not yet available. Therefore, manipulation with an endogenous antioxidant by chemoprotective agents, such as natural products rich in antioxidants or supplementation with traditional antioxidants, has become an alternative modality. A single dose (6 mg/kg) of cisplatin was intraperitoneally injected into rats on the first day of the experiment. Two doses (150 mg/kg and 300 mg/kg) of extracted *Olea europaea* leaves (OEL) were administered to different groups of rats by oral gavage, starting on the 5th day of cisplatin administration until the end of the experiment. Serum biomarkers about kidney function, as well as electrolyte changes, were measured on days 6 and 15 of cisplatin administration. The outcomes of this work showed a significant increase in the serum creatinine, and blood urea nitrogen, and a significant decrease in the potassium level in cisplatin-treated rats was detected. However, administration of a higher dose of the extracted OEL ameliorated cisplatin-induced AKI as evidenced by a significant reduction in the serum creatinine and blood urea nitrogen. In other words, the reduction of toxic parameters means improvement in kidney function. Moreover, the weights of rats were studied in this experiment. It revealed that cisplatin and a higher dose of the extracted OEL prevented weight gain when administered separately and resulted in further reduction in body weight when administered concurrently. Furthermore, cisplatin and OELE caused a significant increase in rats' kidneys when administered separately compared to the control group. However, the weight of the kidney was significantly reduced when cisplatin and a higher dose of OELE were administered concurrently.

The findings of histopathologic examination also confirmed the presence of renal tissue damage in the cisplatin-treated rats. These damages were observed as interstitial inflammation, lymphocyte infiltration, multiple foci of tubulitis, coagulase necrosis, and calcification of the renal tissue. These consequences of cisplatin administration were ameliorated by the administration of different doses of OELE.

The results of the acute toxicity study showed that the crude extract of OEL did not cause physical and behavioral changes as well as mortality during 72 hours of the follow-up toxicity period after administration of 2 g/kg and 5 g/kg of the extracted leaves to different groups. The higher dose during the toxicity study represents more than 15 times the higher tested dose (300 mg/kg) of the extracted leaves. It has been revealed that a good candidate substance for further studies is one with a three times lethal dose (LD50) higher than the minimum effective dose 38, making the doses of the crude extract in this study suitable for further studies.

In the present study, a significant reduction in the body weight of all cisplatin-treated rats was found on the last day of the experiment. The findings of this study are in agreement with other studies that have shown a total body weight reduction after cisplatin administration [41–44]. It seems that weight reduction that occurs after cisplatin administration is attributed to cisplatin-induced anorexia and gastrointestinal disturbances [45, 46]. Connected with weight reduction in cisplatin-treated rats, this study revealed a significant increase in kidneys' weight in this group compared with the control group. This study is in agreement with another finding which shows a correlation between cisplatin treatment and an increase in kidney weight [43, 47, 48].

In this study, the administration of cisplatin significantly caused clinical and histopathological manifestations of kidney dysfunction, including weight loss, infiltration of leukocytes, and necrosis. Moreover, cisplatin administration induced damage in the renal vasculature, leading to declining creatinine clearance. In this study, AKI was manifested as an increase in the serum creatinine level, blood urea nitrogen, and a decrease in the potassium level. This effect is in agreement with several experimental and clinical studies that showed that a single shot of cisplatin generated an overproduction of free radicals, which caused oxidative stress damage and polyunsaturated fatty acid peroxidation in the kidney tissue [43, 49, 50]. Different mechanisms of cisplatin-induced nephrosis have been illustrated in the literature.

The first underline mechanism of cisplatin-induced nephrosis is mostly attributed to the disturbances in the oxidative stress balance, which is manifested by the increase in the reactive oxygen species (ROS) production, especially superoxide anions and hydroxyl radicals and depleted in the enzymatic and nonenzymatic renal antioxidant defense system [51]. Excessive ROS results in destruction of cellular proteins and lipids as well as DNA [52]. However, administration of a higher dose of OELE caused improvement in the kidney function test which might be attributed to restore antioxidant defense capacity in the kidney. The strong antioxidant activity of OEL has been linked to the presence of polyphenolic contents, including oleuropein, hydroxytyrosol, luteolin, and orthodiphenols; as a result, these compounds could prevent the generation of reactive oxygen species [53–55].

The second underline mechanism involved in cisplatin-induced nephrotoxicity is the inflammation of the renal tissue [56]. It has been revealed that cisplatin causes the promotion of a series of inflammatory cytokines, such as IL-1*β* and TNF [57]. However, treatment using OEL prevented the production of inflammatory cytokines, including TNF-*α* and IL-1*β* in many organs, including testicular tissue and gastric tissue [38, 58, 59]. Similarly, Al-Quraishy et al. reported that the gastroprotective effect of OELE was mediated by maintaining the antioxidant activity and restraining inflammation of gastric mucosa by preventing the mobilization of the activated leukocytes and inhibiting nuclear factor-KB (NF-kB) [38]. In line with previous studies, the findings of this study showed that OELE decreased cisplatin-induced tubulointerstitial lesions, lymphocyte infiltration, and coagulative necrosis of the renal tissue. This anti-inflammation effect of OELE has a dose-dependent effect.

The third underline mechanism of cisplatin-induced acute renal damage is renal epithelial apoptosis [60]. The potential molecular mechanisms of cisplatin-induced apoptosis are related to increase the expressions of Bax and p53 genes and suppressing the expression of Bcl-2. However, *Olea europaea* compounds have a beneficial effect against cisplatin-induced nephrotoxicity *in vitro*. For example, in the “GP-293” cell line, incubation of cisplatin-treated cells with oleuropein at a dose of 20 *μ*g/ml could prevent cellular apoptosis by preventing the activation of caspase-3 and reducing Bax: Bcl2 elevation ratio [29]. Another study has shown that treatment with the extracted OEL prevents apoptosis by downregulating Bax and upregulating Bcl-2 in the testicular tissue of cisplatin-treated rats [58].

In this study, serum electrolyte imbalances were detected following cisplatin administration. For example, serum potassium was significantly decreased in cisplatin-treated groups as compared with the control group, and it showed that cisplatin had a cumulative effect on potassium reduction, meaning that the hypokalemic effect of cisplatin on the last day is more prominent compared with the potassium level on the 6th day of the experiment. The higher dose of OELE (300 mg/kg) preserved potassium at a higher level compared with the lower dose of the extracted leaves. This might be related to the restoration effect of the kidney function by the higher dose of OELE, and thus maintained potassium level better than that of the lower dose. The findings of this study are in agreement with other findings that showed a reduction in the potassium concentration in the cisplatin-treated group [42, 47]. On the other hand, a study has shown that hyperkalemia is detected in rats following a single intraperitoneal cisplatin treatment [61].

In this study, a significant improvement in kidney function was observed in the second time frame when compared to the first-time frame. This improvement might result from the ameliorative effect of this extract. Another explanation for this improvement was the washout that happened to the cisplatin concentration from the body after a period of 15 days of administration.

## 5. Conclusion

In conclusion, this study reveals that a single intraperitoneal dose of cisplatin to Wistar rats induces histopathological alterations of the renal tissue, appearing as moderate interstitial inflammation and coagulative necrosis and calcification along with AKI and electrolyte disturbances as evidenced by elevated serum creatinine and blood urea nitrogen and decrease in the serum potassium level. However, the administration of the extracted *Olea europaea* leaves markedly ameliorates cisplatin-induced acute renal injury as explored by improving the renal tissue and reducing creatinine and blood urea nitrogen levels in cisplatin-treated rats, and this effect is obvious with a higher dose of the extract and at the second time point, which proved that the beneficial effect of this extract on renal function is both dose-dependent and time-dependent.

### 5.1. Limitation

This study had several limitations. One limitation was that this study did not assess renal oxidative stress biomarkers, inflammatory markers, and genetic testing. This limitation had consequences on the interpretation of the underline mechanism of the ameliorative effect of the crude extract. In addition, this study evaluated the effect of the crude extract, making the identification of the potential active compound that has the ameliorative effect another limitation. Therefore, further studies are recommended to address this limitation to understand the exact underline mechanism of this effect and to identify the potential active compound that exhibits the ameliorated effect of extracting *Olea europaea* leaves.

## Figures and Tables

**Table 1:** Differences in serum creatinine level at different time points.

Test details (day 6–day 15)	Mean 1 (day 6)	Mean 2 (day 15)	SEM	*P* value
Control	0.458	0.528	0.07138	>0.9999
CIS	3.925	2.3	0.07981	**<0.0001**
CIS/OELE-150	3.7	1.88	0.07981	**<0.0001**
CIS/OELE-300	2.803	1.27	0.07981	**<0.0001**
OELE-150	0.4375	0.625	0.07981	0.1751
OELE-300	0.508	0.694	0.07138	0.1015

CIS: cisplatin; OELE: *Olea europaea* leaf extract; SEM: standard error of the mean. Bold values indicates significant differences.

**Table 2:** Differences in serum urea of the rats at different time points.

Test details (day 6–day 15)	Mean 1 (day 6)	Mean 2 (day 15)	SEM	Adjusted *P* value
Control	38.74	54.98	17.18	0.9287
CIS-	441.8	186.8	19.21	**<0.0001**
CIS/OELE-150	404.5	175.5	19.21	**<0.0001**
CIS/OELE-300	311.5	124	19.21	**<0.0001**
OELE-150	38.45	49.65	19.21	0.9934
OELE-300	30.69	75.46	17.18	0.0973

CIS: cisplatin; OELE: *Olea europaea* leaf extract; SEM: standard error of the mean. Bold values indicates significant differences.

**Table 3:** Effect of OELE and/or cisplatin on the creatinine clearance.

ANOVA table	*F* (5, 18) = 63.82				*P* < 0.0001
Test details	Mean 1	Mean 2	Mean diff.	SE of diff.	Adjusted *P*value
Control group vs. CIS	0.4273	0.11	0.3174	0.02423	**<0.0001**
Control group vs. OELE-150	0.4273	0.3987	0.02862	0.02423	0.8400
Control group vs. OELE-300	0.4273	0.3568	0.07052	0.02285	0.0598
CIS vs. CIS/OELE-150	0.11	0.1206	−0.01068	0.02759	0.9987
CIS vs. CIS/OELE-300	0.11	0.1785	−0.06858	0.02759	0.1801

CIS: cisplatin; OELE: *Olea europaea* leaf extract; SEM: standard error of the mean. Bold values indicates significant differences.

**Table 4:** Differences in serum potassium of the rats at different time points.

Test details (day 6–day 15)	Mean 1 (day 6)	Mean 2 (day 15)	SEM	Adjusted *P* value
Control	6.1	6.56	0.1966	ns
CIS	5.45	5.6	0.2198	ns
CIS/OELE-150	5.225	4.85	0.2198	ns
CIS/OELE-300	5.25	5.25	0.2198	ns
OELE-150	6.35	6.375	0.2198	ns
OELE-300	6.3	6.22	0.1966	ns

CIS: cisplatin; OELE: *Olea europaea* leaf extract; SEM: standard error of the mean.

**Table 5:** Change in body weight between 1st and 15th days of the experiment.

Body weight (day 1–day 15)	Mean 1 (day 6)	Mean 2 (day 15)	% Change	*P* value
Control	130	143.6	+10.5	**0.0054**
CIS	156.3	150	+4	0.5363
CIS/OELE-150	150.3	121.3	−19.3	**<0.0001**
CIS/OELE-300	154	137	−11	**0.0071**
OELE-150	159	170.5	+7.2	**0.0451**
OELE-300	169.4	169.4	0	>0.9999

CIS: cisplatin; OELE: *Olea europaea* leaf extract; SEM: standard error of the mean. Bold values indicates significant differences.

## Data Availability

The data used to support the findings of this study are available from the corresponding author upon request.
